# Structural Insights into Human Peroxisome Proliferator Activated Receptor Delta (PPAR-Delta) Selective Ligand Binding

**DOI:** 10.1371/journal.pone.0033643

**Published:** 2012-05-11

**Authors:** Fernanda A. H. Batista, Daniela B. B. Trivella, Amanda Bernardes, Joyce Gratieri, Paulo S. L. Oliveira, Ana Carolina M. Figueira, Paul Webb, Igor Polikarpov

**Affiliations:** 1 Instituto de Física de São Carlos, Universidade de São Paulo, São Carlos, Sao Paulo, Brazil; 2 Laboratório Nacional de Biociências, Centro Nacional de Pesquisas em Energia e Materiais (CNPEM/ABTLUS) Laboratório Nacional de Biociencias (LNBio), Campinas, Sao Paulo, Brazil; 3 Diabetes Center and Cancer Research Unit, The Methodist Hospital Research Institute, Houston, Texas, United States of America; National Institute for Medical Research, United Kingdom

## Abstract

Peroxisome proliferator activated receptors (PPARs δ, α and γ) are closely related transcription factors that exert distinct effects on fatty acid and glucose metabolism, cardiac disease, inflammatory response and other processes. Several groups developed PPAR subtype specific modulators to trigger desirable effects of particular PPARs without harmful side effects associated with activation of other subtypes. Presently, however, many compounds that bind to one of the PPARs cross-react with others and rational strategies to obtain highly selective PPAR modulators are far from clear. GW0742 is a synthetic ligand that binds PPARδ more than 300-fold more tightly than PPARα or PPARγ but the structural basis of PPARδ:GW0742 interactions and reasons for strong selectivity are not clear. Here we report the crystal structure of the PPARδ:GW0742 complex. Comparisons of the PPARδ:GW0742 complex with published structures of PPARs in complex with α and γ selective agonists and pan agonists suggests that two residues (Val312 and Ile328) in the buried hormone binding pocket play special roles in PPARδ selective binding and experimental and computational analysis of effects of mutations in these residues confirms this and suggests that bulky substituents that line the PPARα and γ ligand binding pockets as structural barriers for GW0742 binding. This analysis suggests general strategies for selective PPARδ ligand design.

## Introduction

It is important to develop rational strategies for development of highly selective nuclear hormone receptor (NR) ligands; homology between closely related family members means that drugs which activate particular NRs can cross-react with others, often triggering undesirable side effects. There are three peroxisome proliferator activated receptor (PPAR) subtypes termed PPARβ/δ (hereafter δ), PPARα and PPARγ with different expression profiles and actions [1]. PPARδ activation improves overall metabolic profile. While no PPARδ agonists are yet approved for human use, they have been shown to enhance fatty acid oxidation in skeletal muscle, reduce serum triglycerides, increase serum high density lipoprotein (HDL) cholesterol and stimulate aspects of reverse cholesterol transport, improve glucose homeostasis, and trigger thermogenesis and weight loss [2], [3], [4]. Additionally, PPARδ ligands even enhance metabolic benefits of exercise training and can act as an exercise mimetics in their own right. Whereas agonists that activate other PPARs exert beneficial effects, these actions are tempered by deleterious side effects. PPARγ agonists (thiazolidinediones, TZDs) are potent insulin sensitizers [5], [6] but cause edema, gain in fat mass, increased bone fractures and elevated risk of heart attack which have led to restrictions in their use. Fibrates that activate PPARα [6] reduce serum triglycerides and increase HDL but PPARα agonists are carcinogenic in rodents. Dual specificity ligands (glitazars) that simultaneously activate PPARα and PPARγ elicit significant improvements in insulin sensitivity and atherogenic serum lipid profiles in humans, but were discontinued because of cardiovascular events and increased death rate, carcinogenicity in rodents, liver toxicity and kidney damage. Current indications suggest that desirable PPARδ agonists should not cross-react with other PPARs.

PPARs exhibit complex ligand binding modes. PPAR C-terminal ligand binding domains (LBDs) are 60–70% homologous [7] with large (≈1300Å^3^) Y-shaped ligand binding pockets (LBPs) composed of three sub-arms (Arms I, II and III) that display significant homology between the subtypes. Arm I is predominantly polar, well conserved and includes residues that line C-terminal activation helix 12 (H12rs) [8], [9], [10]. Arms II and Arm III are predominantly hydrophobic and less well conserved among PPARs [9], [11]. All three PPARs bind a variety of natural and synthetic ligands, none of which completely fills the LBP and PPAR ligands can adopt different binding modes [9]. Many agonists, however, conform to a standard pharmacophoric model [12] in which ligands comprise a hydrophilic head group that binds Arm I and a hydrophobic tail that binds Arm II and/or Arm III.

**Figure 1:**
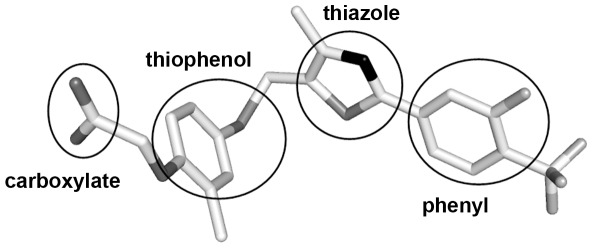
Three-dimensional structure of PPARδ-selective agonist GW0742, as found in our hPPARδ:GW0742 crystal structure (PDB id 3TKM). Typical structural features of PPAR agonists are displayed. Carbon, fluoride, sulfur, oxygen and nitrogen atoms are colored white, light grey, grey, dark grey and black, respectively.

GW0742 (Fig. 1) was developed using standard medicinal chemistry and conforms to the pharmacophoric model of PPAR ligands, yet displays 300–1,000 fold selectivity for PPARδ versus other PPARs [13] and full PPARδ agonist actions in cell culture and animal models [14], [15], [16], [17]. Presently, however, the structural basis for this high selectivity is not obvious. Whereas X-ray structures of PPARδ in complex with PPARδ-specific partial agonists are reported and reveal ligand binding within parts of Arms II and III far from H12, X-ray structures of PPARδ in complex with GW0742 or other PPARδ selective agonists are not publicly reported.

Here, we report the resolution of the structure of the PPARδ LBD in complex with GW0742 to gain insights into selective binding of this ligand and methods to improve PPARδ-selective binding of agonists that conform to the standard pharmacophoric model. Comparisons of the docking mode of this GW0742 with those of highly hPPARα and hPPARγ selective agonists with their respective receptors and a pan agonist with all three PPARs coupled to mutational and computational analysis of effects of PPARδ mutants identifies two LBP residues (Val312 and Ile328) that are crucial for specificity, pinpointing regions of the LBP that could be explored in new ligand development.

## Results and Discussion

### hPPARδ-LBD:GW0742 Complex Structure

**Figure 2:**
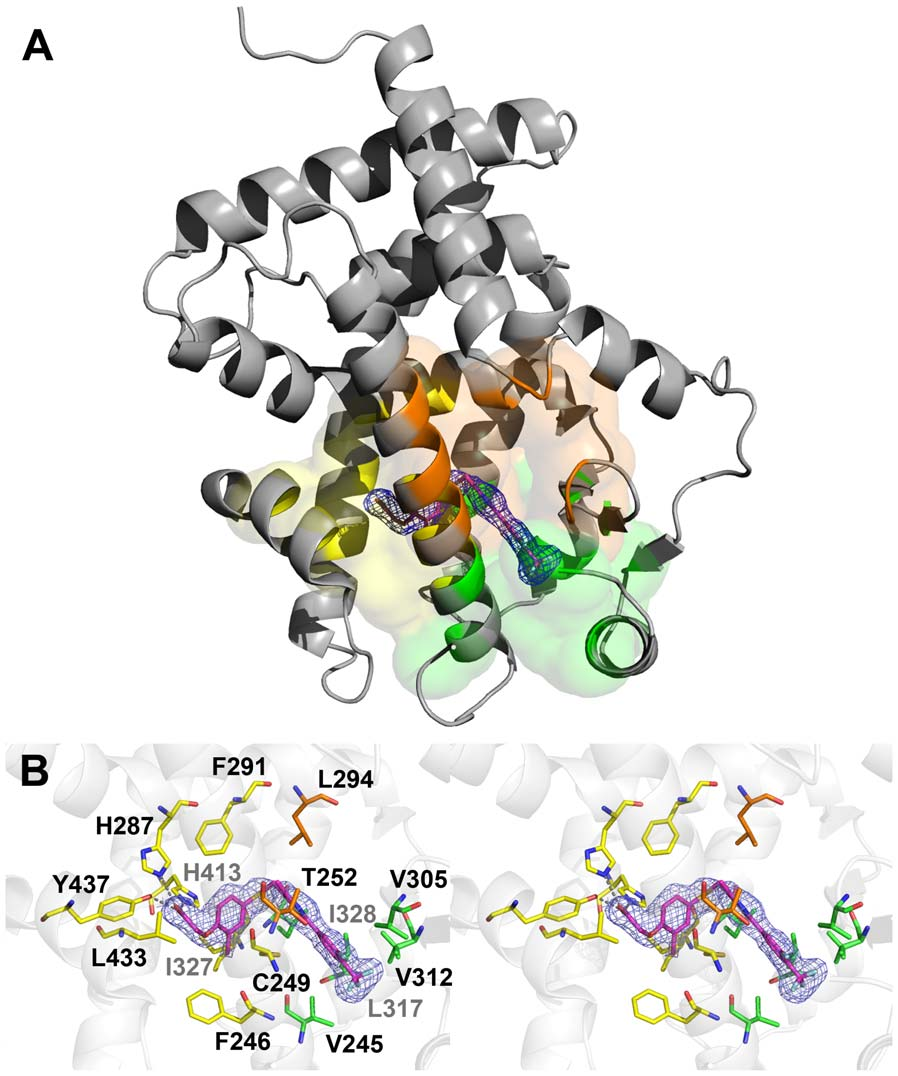
Crystallographic structure of the complex hPPARδ-LBD:GW0742. *(A)* The ligand (magenta sticks) occupies the PPARδ-LBD (grey cartoon) and performs interactions with residues belonging to the arm I (yellow), arm II (green) and arm III (orange). *(B)* Stereo view of the binding site, showing the electron density calculated for the ligand (omit map, contoured at σ = 1.0) and the PPARδ residues that stabilize the ligand. Polar interactions between hPPARδ-LBD and the GW0742 ligand are shown as dashed lines. Nitrogen, oxygen, sulfur and fluoride atoms are colored blue, red, yellow and light blue, respectively. The residues from arms I, II and III are colored in yellow, green and orange, respectively. Figures were generated with the Pymol software (Schrödinger).

The crystal structure of hPPARδ-LBD with GW0742 was determined in the P2_1_2_1_2_1_ space group, at 1.95 Å resolution (Fig. 2A). The final model consists of a monomer in the asymmetric unit, composed of residues Gln171 to Tyr441 (hPPARδ numbering). One molecule of GW0742, 185 water molecules and one glycerol molecule were also resolved in the structure. All protein residues occupy favorable regions of the Ramachandran plot; data collection statistics are given in Table 1. Overall folding resembles previous PPARδ LBD structures and is not further described.

**Table 1:** Crystallographic data collection statistics.

Parameter	
Wavelenght (Å)	1.46
Space Group	P2_1_2_1_2_1_
Unit Cell Dimensions (Å)	35.466 41.766 96.287
Resolution Range (Å)	24.4 (1.95)
Reflections at working set	19134 (2511)
Reflections at test set	978 (135)
Redundancy	5.8 (4.7)
Completeness (%)	99.28 (99.1)
I/δ	17.3 (2.6)
Rfree	24.5 (30.2)
Rfactor	19.5 (25.6)
RMSD bond lengths (Å)	0.004
RMSD bond angles (degrees.)	1.006
Average B-factor	24.85
Ramachandran outliers	0/303

Values in parentheses indicate the high-resolution shell.

GW0742 occupied the Y-shaped LBP and adopted a position predicted by the pharmacophoric model of PPAR ligands [8], [9], [10], [18] (Fig. 2B). The hydrophilic head group interacts with arm I and the hydrophobic tail, comprising the thiazole and the fluorine substituted phenyl ring, is positioned mostly in arm II. The linker connecting the head and tail groups lies close to H3 (Fig. 2B). In total, GW0742 made 29 ligand interactions with PPARδ pocket, including three polar interactions and 26 apolar interactions (Table S1).

Polar interactions mostly involve the ligand hydrophilic head group and residues in Arm I and appear similar to other PPAR agonists with their respective PPARs [9]. By analogy, these interactions are probably responsible for maintaining the locked agonist conformation of activation helix 12. One ligand carboxylate oxygen engages in hydrogen bonds with the side chains of residues His413 (helix 10/11) and Tyr437 (helix 12) - Figure 2B. The other carboxylate oxygen contacts the His287 side chain from PPARδ helix 7.

Apolar interactions involved residues in all three Arms. In Arm I Phe246, Phe291, His413, Ile327, Leu433 and Cys249 side chains contact ligand. In arm II, Val245, Val305, Val312, Leu317 and Ile328 side chains bind ligand and two residues that lie within Arm III, Thr252 and Leu294, are also engaged in ligand contact.

**Figure 3:**
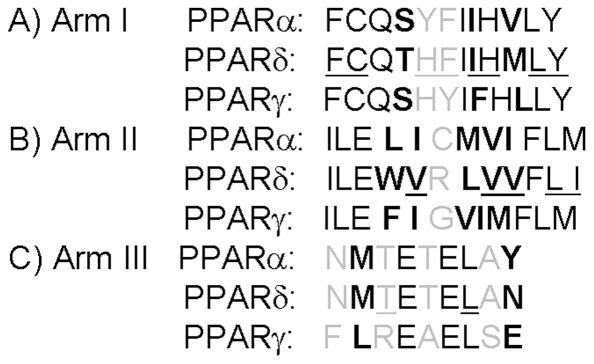
Alignment of amino acid residues forming the binding site of the different human PPAR isotypes. Residues placed in arm I (A), arm II (B) and arm III (C) are shown. Residues involved in the hPPARδ-LBD:GW0742 interactions are underscored. Residues in black, bold and gray represent identical residues, residues with same chemical character and residues with different chemical character, respectively.

We were not able to discern any GW0742 contacts with amino acids that were completely unique to PPARδ and could account for selective ligand binding (Fig. 3) [18]. Of 12 Arm I amino acids (Fig. 3A); eight (Phe246, Cys249, His287, Phe291, Ile327, His413, Leu433 and Tyr437) contact GW0742. Of these, His287, Phe291 and Ile327 vary between PPARs and none are exclusive to PPARδ; Phe291 and Ile327 are conserved in PPARα and His287 is conserved in PPARγ. Of 12 Arm II residues (Fig. 3B), five (Val245, Val305, Val312, Leu317 and Ile328) are involved in ligand contact. Of these; Leu317 is identical in all subtypes and there are conserved substitutions at the other four positions. Of nine Arm III residues (Fig. 3C), only two contact ligand; Leu294 is conserved in the three PPAR subtypes and Thr252 is conserved in PPARα with a non-conserved substitution in PPARγ.

### Potential Steric Hindrance to GW0742 Binding in PPARα and γ

**Figure 4:**
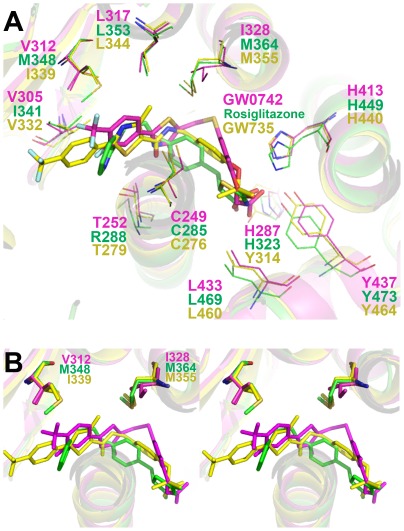
Crystallographic structure superposition of selective ligands to each PPAR isotype. Helices from PPAR are shown as yellow, magenta and green cartoons for PPARα, δ and γ, respectively. The α selective ligand, GW735 (PDBid: 2P54), the δ selective ligand, GW0742, and the γ selective ligand, rosiglitazone (PDBid: 2PRG), are shown as yellow, magenta and green sticks, respectively. Oxygen, nitrogen, sulfur and fluoride atoms are shown in red, blue, yellow and light blue, respectively. A) Upper vision of the binding site. B) Stereoscopic view of the PPAR binding sites, highlighting the importance of Val312 and Ile328 in GW0742 accommodation and GW735 and rosiglitazone displacement, presumably due to the presence of bulky substitutions. Ligands GW735, GW0742, rosiglitazone are painted in yellow, magenta and green, respectively.

We next compared the PPARδ:GW0742 structure with analogous structures of PPARα and γ LBDs in complex with representative selective agonists (GW735 and Rosiglitazone) and the three PPARs with a pan agonist, indeglitazar (PDB ids: 2P54 [19], 2PRG [20] and 3ET2, 3ET3 and 3ET1, respectively [21]. All four ligands conform to the standard PPAR ligand pharmacophoric model [22] and adopt a similar position in the pocket (Fig. 4). However, GW0742 binding exhibited two features that were unique. First, the linker group is displaced from H3 relative to other PPAR subtype selective ligands (Fig. 4A). This shift was also seen in the PPARδ structure with the non-selective agonist indeglitazar (not shown), suggesting that it does not account for selectivity. More interestingly, the GW0742 hydrophobic tail occupies the entrance to Arm II, unlike GW735 and Rosiglitazone tails which are directed towards Arm III between the helices 3 and 2̀ (Fig. 4A and B).

Comparison of amino acids that form the PPARδ Arm II entrance with equivalent regions of PPARα and PPARγ revealed two substitutions which could potentially form barriers to GW0742 binding and could block access to PPARγ and PPARα Arm II; δVal312 is replaced by bulkier side chains αIle339 and γMet376 and δIle328 is substituted by the bulkier methionine in both PPARs (αMet355 and γMet392) (Fig. 4B). Other nearby substitutions do not exhibit similar potential to block GW0742 binding. Some introduce similarly sized amino acids (δHis287/αTyr314/γHis351; δPhe291/αPhe318/γTyr355; δVal245/αIle272/γIle309 and δVal305/αVal332/γIle369). Nearby PPARγ-specific substitutions (δIle327/αIle354/γPhe391 and δThr252/αThr279/γArg316) introduce residues with flexible side chains that are not likely to block GW0742 binding; γPhe391 contacts Rosiglitazone (PDB id 2PRG), but faces away from ligand in the PPARγ:Indeglitazar structure (PDB id 3ET3) (Figure S1) and γArg316 faces away from both ligands.

### Site Directed Mutagenesis Confirms Key Roles for δVal312 and δIle328 in GW0742 Binding

**Figure 5:**
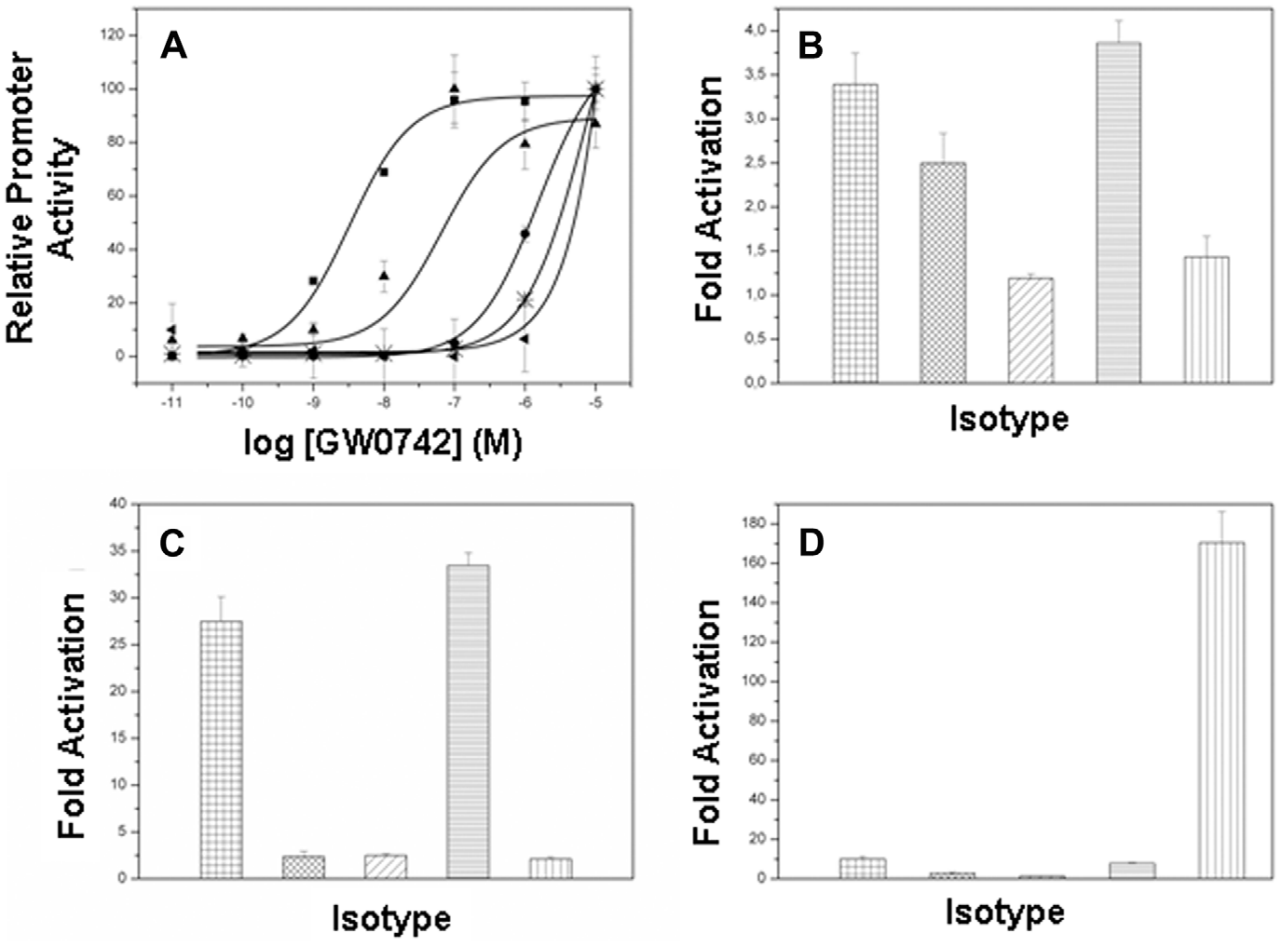
PPAR transactivation assays. PPAR activation induced by *(A)* the δ-selective agonist GW0742; *(B)* the pan-agonist benzafibrate; *(C)* the α-selective agonist GW7647 and *(D)* the γ-selective agonist rosiglitazone. All data were normalized by the level of *Renilla* luciferase activity. ▪/**#** wtPPARδ, ▴/xx PPARδVal312Met, •/**//**PPARδIle328M, <$>\vskip -1\scale 80%\raster="rg1"<$>/ = PPARα and ◂/<$>\vskip -1\scale 70%\raster="rg2"<$>PPARγ.

To determine whether δVal312 and δIle328 are important for PPARδ selective activation by GW0742, we introduced Met substitutions at both positions: PPARδ-LBD/Val312Met and PPARδ-LBD/Ile328Met and determined effects of mutations on responses to different ligands. As expected [13], GW0742 was a potent activator of PPARδ (EC_50_ = 3.25 nM) relative to PPARα or PPARγ; it was not possible to derive accurate EC_50_ values for the latter curves. Both PPARδ mutants displayed similar levels of activation at very high GW0742 concentrations, but EC_50_ values were greatly increased relative to wild type receptor, indicative of reduced potency (Fig. 5A). Half-maximal responses were one order of magnitude higher (66.0 nM) for PPARδIle328Met relative to wild type receptor and EC_50_ values for PPARδVal312Met mutant were even higher, it was not possible to achieve an adequate estimate of EC_50_ values similar to wild type PPARα and PPARδ.

The met substituents did not completely change overall PPARδ ligand binding profile. The pan-PPAR agonist benzafibrate [23]; activated PPARα, PPARδ and PPARγ with descending efficacy (Fig. 5B) and PPARδ Val312Met activation was about 2.5 fold, similar to wild type PPARδ and PPARδIle328Met was similar to that of PPARγ (1.5 times of activation). Neither met substituent enhanced activation by the PPARα selective agonist GW7647 (Fig. 5C) or the PPARγ selective agonist rosiglitazone (Fig. 5D). Thus, the presence of bulky residues at positions 312 and 328 reduces PPARδ activation by GW0742 but does not permit PPARδ activation by ligands that bind other subtypes.

### Docking and Molecular Dynamics Simulation

We next modeled PPARδ-LBD mutants, docking the ligand inside the structures. After docking, energy minimization and molecular dynamics simulation, all the PPARs (α, γ, δ and the mutants δVal312M and δIle328M) showed accommodation of their main chains, with trajectory root mean square deviation (RMSDs) ranging from 1.3 Å to 1.65 Å. Docking analysis revealed that for all PPARs, GW0742 was able to accommodate itself in the ligand binding pocket, but considerable conformational changes of the side chains, which corresponds to the Val312 and Ile328 substitutions, and also in the ligand were observed (data not shown).

Analysis of RMSDs of Met312 and Ile328, after simulations, shows that PPARδ presents smaller conformational changes in comparison to the other PPARs (Table 2), clearly revealing necessity of large side chain adjustments by PPARα, γ and the mutants, in order to accommodate GW0742 ligand.

**Table 2:** RMSD values of the residues δMet312, δIle328 and its corresponding residues from PPAR α, γ and mutants after GW0742 docking and molecular dynamic simulations.

model	RMSD of δMet312 position(Å)	RMSD of δIle328 position (Å)
PPARδ	0.3	0.6
PPARα	0.6	1.1
PPARγ	1	1.8
PPARδ V312M	0.9	0.7
PPARδ I328M	0.3	0.9

In summary, we have solved the PPARδ-LBD structure in complex with GW0742, a high potent and selective PPARδ agonist. The ligand follows the binding model predicted to other PPAR ligands based on the same pharmacoforic groups. The carboxylate group occupies arm I of the binding pocket while the hydrophobic tail occupies arm II. Comparison of the structures of the three PPARs isotypes with agonists allowed us to observe some subtle differences that could explain the isotype delta ligand selectivity to GW0742. Specifically, the hydrophobic tail of GW0742 occupies part of Arm II, unlike equivalent PPARα and PPARγ agonists which dock into Arm III and we propose that the presence of two residues in PPARδ-LBD, Val312 and Ile328, is intimately related with selectivity. Here, both of these residues are replaced by amino acids with bulkier side chains in PPARα and PPARγ, and it is likely that these would occlude the entrance to ArmII in the context of these PPAR subtypes and prevent the GW0742 hydrophobic tail from docking into its preferred position. To validate this hypothesis, we performed two single point mutations, Val312Met and Ile328Met, and conducted cell activation assays and docking analyses of PPAR isotypes and mutants using selective ligands for each isotype and confirmed that introduction of substituents that resemble other PPARs at these positions reduces activation of PPARδ by GW0742 but not other non-selective PPAR ligands. Our results indicate that ligands carrying short linkers and large and rigid hydrophobic tails find difficulties in being accommodated into PPARα and PPARγ arm II, probably as a consequence of the bulky amino acid substitution found in these isotypes. We propose that this hypothesis brings some light to the understanding of the molecular basis of PPAR selective ligands mode of interaction and may be helpful in further rational design of PPAR selective agonists.

Our results agree with previous studies which link effects of amino acid substitution in PPARs binding sites upon ligand binding to the binding site shape, which, in turn, limits ligand entry and accommodation [8], [9], [10], [18], [24]. PPARδ presents the smaller arm I as a consequence of the presence of Met417 in the place of the Val residue present in PPARα, what explains the relative low affinity of this isotype for some fibrates and other ligands with large groups linked to the hydrophilic head [18], [24]. In the same direction, the presence of Tyr344 in PPARα arm III reduces the size of the binding site entrance, causing steric restrictions to ligand entry [8]. Substitutions in arm II were mainly related to change the accommodation of the main hydrophobic part of the ligands [8], [18]. This mode of selectivity is very different from that of other NRs, such as thyroid hormone and estrogen receptors, where selectivity often relates to enhanced contacts between ligand and specific amino acids within the pocket. It will be important to understand the rules that link pocket shape to ligand position in PPARs to better develop new selective ligands.

## Methods

### Protein Expression and Purification

The human PPARδ LBD plasmid (amino acids 171–441) with cDNA inserted into pET15 vector (Novagen, USA) was transformed in BL21(DE3) *Escherichia coli*. Protein expression was performed in LB culture, induced with 1 mM IPTG, at 18°C for 12 h. Cells were harvested and ressuspended in a 20 ml of buffer A (20 mM Hepes pH 7.5, 300 mM NaCl, 5% glycerol, 10 mM β-mercaptoethanol, 10 mM PMSF and 250 µg/mL lysozyme) per liter of culture. The lysate was sonicated, clarified by centrifugation and loaded onto a Talon Superflow Metal Affinity Resin (BD Biosciences Clontech, Palo Alto, CA), and eluted with an imidazol gradient (0–300 mM). The fractions containing the purified protein were pooled and washed, using centrifugal concentrators (Amicon, 10 MW cutoff), to remove imidazol. The His-tag was cleaved with trombin (7 U/mg), at 18°C, overnight. Protein purity was checked by Coomassie blue-stained SDS-PAGE. Protein concentrations were determined using the Bradford dye assay (Bio-Rad, Hercules, CA).

### Crystallization

Protein buffer was changed to 20 mM Hepes (pH 7.5), 500 mM ammonium acetate, 10 mM β- mercaptoethanol, according to [8]. Prior to crystallization, PPARδ-LBD (256 µM) was incubated for 4 h with GW0742 (Tocris Bioscience) (1∶4 protein:ligand molar ratio) in DMSO (DMSO final concentration equals to 5%), at 4°C. The sitting-drop vapor diffusion method was used, with drops containing 2 µl of protein:ligand complex, 0.5 µl of the detergent n-Octyl-b-D-thioglucoside and 2.5 µl of the reservoir solution made of 14% (w/v) polyethylene glycol (PEG) 8000, 200 mM KCl, 40 mM *bis*-Tris-propane (pH 9.5), 6% propanol, 1 mM CaCl_2_. hPPARδ-LBD:ligand co-crystals were grown at 18°C and appeared after 3 days, showing a well-defined geometric form.

### Data Collection, Model Refinement and Analysis

Crystals were transferred to a cryo-protecting solution, containing the well solution plus 10% glycerol, and immediately flash cooled to 100 K in a nitrogen stream prior to data collection. The X-ray diffraction data collection was performed at the MX-2 beamline of the Brazilian National Synchrotron Light Laboratory (LNLS, Campinas, Brazil) [25] using synchrotron radiation of wavelength 1.459 Å to optimize crystal diffraction efficiency and the synchrotron-radiation flux of the LNLS storage ring [26]. The diffraction images were registered on a MAR225 mosaic detector, with an oscillation of 1° per image. Data reduction was performed using HKL200/Scalepack package [27].

The X-ray structure of PPARδ-LBD (PDB ID: 3ET2) [21] was used as an initial model for molecular replacement using the program PHASER [28]. The protein atomic model was improved through alternated cycles of real space refinement using COOT [29] and maximum likelihood minimization using PHENIX [30]. Ligand and solvent molecules were included in the last steps of refinement.

Protein:ligand contacts were analyzed using the Ligplot software [31], followed by visual inspection using the program COOT [29]. A hydrogen bond distance cutoff of 3.4 Å was applied. Superposition of different PPAR crystal structures was performed with the Superpose software [32] and analyzed using the Pymol software [33].

### PDB Accession Code

The atomic coordinates and structure factors of the hPPARδ-LBD:GW0742 crystal complex reported here are deposited in the Protein Data Bank under code 3TKM.

### Site Directed Mutagenesis and Transactivation Assay

Mutations in the hPPARδ-LBD were introduced by PCR in an existing vector PPARδGAL4 [34] with overlapping of mutated primers and vector using the QuikChange site-directed mutagenesis kit (Stratagene). All mutated constructs were verified by sequencing. The reporter plasmid pGRE-LUC (GAL4 responsive element, *Firefly* luciferase reporter vector) and PPARδ LBDGal4 inserted in pBIND (Promega). The pRL-TK, that contains *Renilla* luciferase, was purchased from Promega (*Dual-Luciferase Report Assay system* Promega, Madison, WI).

HepG2 cells were cultured in Dulbecco’s Modified Eagle’s Medium (DEMEM) supplemented with 10% heat-inactivated fetal bovine serum, 2 mM glutamin, 50 UI/mL penicillin/streptomycin under 95% air and 5% CO_2_ at 37°C. For transactivation assays, the cells were removed by trypsinization and replated in 24 wells plate at density of 1,2×10^5^ cells/well. Cell transfections were performed using FuGENE 6 transfection reagent (Roche, Swiss) with 100 ηg of plasmids containing wild-type PPARα,δ ou γ-LBD or PPARδ-LBD mutants, DBD Gal-4, 50 ηg of luciferase reporter plasmid and 1 ηg of *Renilla* luciferase plasmid per well. Cells were treated with different concentrations of agonists of PPARα - GW7647, PPARδ - GW0742 and PPARγ - Roziglitazone, in triplicate 24 h after transfection and incubated for additional 24 h. Cell lysates were prepared and luciferase assay was performed using the *Dual-Luciferase Report Assay system* (Promega, Madison, WI*),* following manufacturer instructions. Light emission was measured by integration over 5 seconds of reaction in a Safire luminescent counter (Tecan, Tecan US, NC, USA). Firefly luciferase activity was normalized by the level of *Renilla* luciferase activity, as recommended by manufacturers *Dual-Luciferase Report Assay system*. Data were fitted using a sigmoidal dose-response function with corresponding EC50 determination according to GraphPad Prism software (version 5.0).

### Docking and Molecular Dynamics Simulation

The molecular complexes for PPAR α, γ, mutants and GW0742 were built using the ligand conformation obtained from crystallographic structure of PPARδLBD:GW0742 complex (PDBid 3TKM). PPAR α and γ LBD structures (PDBid 3ET1 and 3ET3 respectively) were superposed to PPARδ complex and coordinates of the ligand were copied to the PPAR α and γ structures. Mutant PPARδ-LBD models V312M and I328M were built using the YASARA software. All structures were submitted to energy minimization and molecular dynamics simulation using YASARA. For that, all hydrogen atoms and other missing atoms from the model were created using force field parameters, obtained from YAMBER3. A simulation box was defined at 15 Å around all atoms of each complex. Protonation was performed based on the pH 7. Cell neutralization was reached filling the box with water molecules and Na^+^/Cl^−^ counter ions. A short molecular dynamics (MD) simulation was performed for the solvent adjust, deleting water until the density of 0.997 g/ml was reached. A short steepest descent energy minimization was carried until the maximum atom speed dropped below 2200 m/s. Then 500 steps of simulated annealing were performed with a temperature of 0 K. Finally, a 4 ns (nanosecond) simulation at 298 K and a non-bonded cutoff of 7.86 A was performed. A snapshot was saved every 25 ps (picosecond). Simulation time was adjusted to stabilize the contacts between protein and ligand.

## Supporting Information

Figure S1 Observation of phenylalanine flexibility on PPARγ structures. Superposition of the γ-selective ligand rosiglitazone (green stick), pan-agonist ligand indeglitazar (blue sticks) and the γPhe391 residue from the respective crystallographic structures for PDB id 2PRG (green lines) and 3ET3 (green lines). Helix 3 is shown as a blue and green cartoon. Oxygen, nitrogen, sulfur and fluoride atoms are shown in red, blue, yellow and light blue, respectively.
(DOC)

Table S1 Atoms involved in interactions between the GW0742 ligand and hPPARδ-LBD, as found in our hPPARδ-LBD: GW0742 crystal structure.
(DOC)
