# Supplementary material for: Structural Insights into Human Peroxisome Proliferator Activated Receptor Delta (PPAR-Delta) Selective Ligand Binding
Source: PLoS One. 2012 May 11;7(5):e33643. doi: 10.1371/journal.pone.0033643 (PMC3350516; doi:10.1371/journal.pone.0033643)
Supplement: Figure S1 — Observation of phenylalanine flexibility on PPARγ structures. Superposition of the γ-selective ligand rosiglitazone (green stick), pan-agonist ligand indeglitazar (blue sticks) and the γPhe391 residue from the respective crystallographic structures for PDB id 2PRG (green lines) and 3ET3 (green lines). Helix 3 is shown as a blue and green cartoon. Oxygen, nitrogen, sulfur and fluoride atoms are shown in red, blue, yellow and light blue, respectively. (DOC) [file pone.0033643.s001.doc]

**SUPPORTING INFORMATION**

**STRUCTURAL INSIGHTS INTO HUMAN PEROXISOME PROLIFERATOR ACTIVATOR RECEPTOR δ (PPARδ) SELECTIVE LIGAND BINDING**

**Batista, Fernanda A.H.; Trivella, Daniela B. B.; Bernardes, A.; Gratieri, Joyce; Oliveira, Paulo. S. L., Figueira, Ana Carolina M.; Webb, Paul; Polikarpov, Igor**


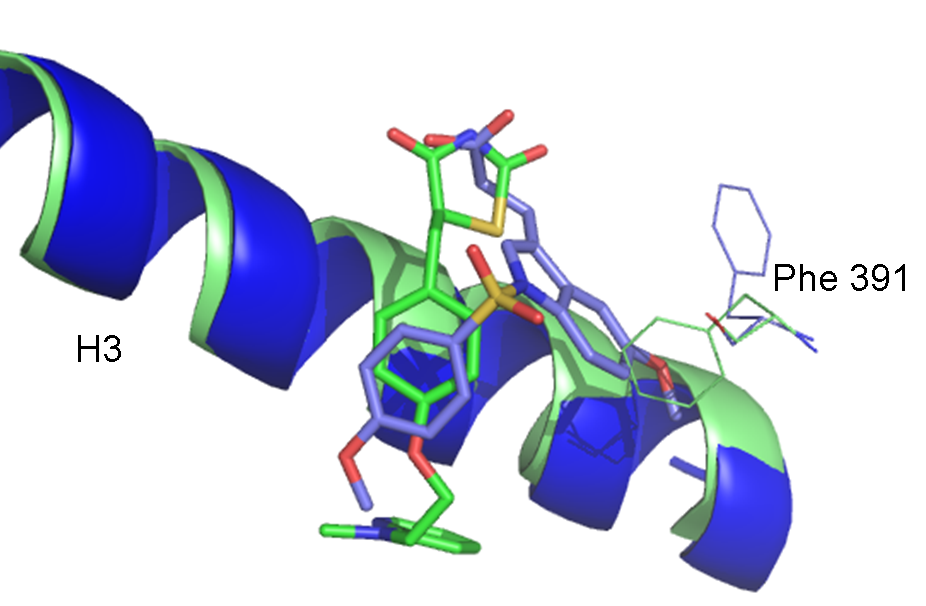


**Figure S1:** Observation of phenylalanine flexibility on PPARγ structures. Superposition of the γ-selective ligand rosiglitazone (green stick), pan-agonist ligand indeglitazar (blue sticks) and the γPhe391 residue from the respective crystallographic structures for PDB id 2PRG (green lines) and 3ET3 (green lines). Helix 3 is shown as a blue and green cartoon. Oxygen, nitrogen, sulfur and fluoride atoms are shown in red, blue, yellow and light blue, respectively.
