# Supplementary material for: Structural Insights into Human Peroxisome Proliferator Activated Receptor Delta (PPAR-Delta) Selective Ligand Binding
Source: PLoS One. 2012 May 11;7(5):e33643. doi: 10.1371/journal.pone.0033643 (PMC3350516; doi:10.1371/journal.pone.0033643)
Supplement: Table S1 — Atoms involved in interactions between the GW0742 ligand and hPPARδ-LBD, as found in our hPPARδ-LBD: GW0742 crystal structure. (DOC) [file pone.0033643.s002.doc]

**SUPPORTING INFORMATION**

**STRUCTURAL INSIGHTS INTO HUMAN PEROXISOME PROLIFERATOR ACTIVATOR RECEPTOR δ (PPARδ) SELECTIVE LIGAND BINDING**

**Batista, Fernanda A.H.; Trivella, Daniela B. B.; Bernardes, A.; Gratieri, Joyce; Oliveira, Paulo. S. L., Figueira, Ana Carolina M.; Webb, Paul; Polikarpov, Igor**

**Table S1:** Atoms involved in interactions between the GW0742 ligand and hPPARδ-LBD, as found in our hPPARδ-LBD: GW0742 crystal structure .

| *Atom* | | *Ligand* | | *Atom* | | *Residue* | | *Bind* | | Length | |
| --- | --- | --- | --- | --- | --- | --- | --- | --- | --- | --- | --- |
| CAO | GW0742 | | CD2 | | LEU | | 433 | | Apolar | | 3.65 |
| CBA | GW0742 | | CE1 | | HIS | | 413 | | Apolar | | 3.69 |
| CAK | GW0742 | | CE1 | | HIS | | 413 | | Apolar | | 3.66 |
| CAI | GW0742 | | CE1 | | HIS | | 413 | | Apolar | | 3.75 |
| CAV | GW0742 | | CE1 | | HIS | | 413 | | Apolar | | 3.85 |
| CAM | GW0742 | | CD1 | | ILE | | 328 | | Apolar | | 3.54 |
| SAS | GW0742 | | CD1 | | ILE | | 328 | | Apolar | | 3.33 |
| SAT | GW0742 | | CD1 | | ILE | | 328 | | Apolar | | 3.44 |
| CAM | GW0742 | | CG2 | | ILE | | 327 | | Apolar | | 3.40 |
| CAL | GW0742 | | CD2 | | LEU | | 317 | | Apolar | | 3.90 |
| CBE | GW0742 | | CG1 | | VAL | | 312 | | Apolar | | 4.63 |
| CBE | GW0742 | | CG2 | | VAL | | 312 | | Apolar | | 3.74 |
| CAY | GW0742 | | CG1 | | VAL | | 305 | | Apolar | | 3.54 |
| CAP | GW0742 | | CD2 | | LEU | | 294 | | Apolar | | 3.44 |
| CAI | GW0742 | | CE1 | | PHE | | 291 | | Apolar | | 3.80 |
| CAB | GW0742 | | CG2 | | THR | | 252 | | Apolar | | 3.70 |
| SAT | GW0742 | | SG | | CYS | | 249 | | Apolar | | 3.67 |
| CBD | GW0742 | | SG | | CYS | | 249 | | Apolar | | 3.55 |
| CAZ | GW0742 | | SG | | CYS | | 249 | | Apolar | | 3.60 |
| CAJ | GW0742 | | SG | | CYS | | 249 | | Apolar | | 3.60 |
| CAA | GW0742 | | CB | | CYS | | 249 | | Apolar | | 3.44 |
| CAV | GW0742 | | CB | | CYS | | 249 | | Apolar | | 3.52 |
| CAM | GW0742 | | CB | | CYS | | 249 | | Apolar | | 3.84 |
| CAA | GW0742 | | CE1 | | PHE | | 246 | | Apolar | | 3.50 |
| CAA | GW0742 | | CD1 | | PHE | | 246 | | Apolar | | 3.52 |
| CAL | GW0742 | | CG1 | | VAL | | 245 | | Apolar | | 3.87 |
| OAD | GW0742 | | OH | | TYR | | 437 | | Polar | | 2.70 |
| OAD | GW0742 | | NE2 | | HIS | | 413 | | Polar | | 2.76 |
| **OAC** | GW0742 | | NE2 | | HIS | | 287 | | Polar | | 2.80 |
